# Sugammadex and amino acid infusion can contribute to safe anesthetic management of variegate porphyria

**DOI:** 10.1186/s40981-018-0187-9

**Published:** 2018-06-18

**Authors:** Yoshitaka Aoki, Kazuyuki Atsumi, Makiko Kora, Naoko Koh, Junichiro Yokoyama

**Affiliations:** Department of Anesthesiology, Shizuoka General Hospital, 4-27-1 Kita-Ando, Aoi-Ku, Shizuoka 420-8527 Japan

**Keywords:** Porphyria, General anesthesia, Sugammadex, Amino acid infusion

## Abstract

**Background:**

Variegate porphyria (VP) is an inherited type of porphyria characterized by cutaneous manifestations and/or acute neurovisceral attacks. We report successful anesthetic management of VP.

**Case presentation:**

A 66-year-old woman with VP was scheduled to undergo distal pancreatectomy for pancreatic cancer. Medical history was unremarkable except for sudden onset of abdominal pain that occurred every few months, possibly due to VP. There was no abnormality in laboratory data except for a mild increase in creatinine levels. General anesthesia was induced and maintained with total intravenous anesthesia using propofol, remifentanil, and rocuronium. Blood glucose levels were measured every hour and glucose administered to prevent hypoglycemia. Amino acids were also infused to treat hypothermia. Upon completion of distal pancreatectomy, sugammadex was administered to reverse neuromuscular blockade. She was neurologically intact and discharged on postoperative day 15 with no acute attack.

**Conclusions:**

Sugammadex and amino acids can be used safely in patients with VP.

## Background

Variegate porphyria (VP) is an autosomal-dominant disease caused by mutation of the protoporphyrinogen oxidase gene (*PPOX*) [1]. It has a worldwide prevalence of 1/300,000 individuals. VP is characterized by cutaneous manifestations and/or acute neurovisceral attacks such as vomiting, motor neuropathy, paresis, seizure, agitation delirium, psychosis, electrolyte abnormalities, and renal failure [2]. Avoidance of possible triggers of attacks, such as hypoglycemia and hypothermia, during the perioperative period is very important. Several anesthesia-related agents should also be avoided.

Because of its rarity, much remains to be clarified about the anesthetic management of patients suffering from VP. In this case, we selected two agents used in routine anesthesia: sugammadex (to reverse the actions of the muscle relaxant applied during anesthesia) and an amino acid infusion (to increase body temperature). Sugammadex has been reported in only one VP case [3], and amino acid infusion has never been reported in a VP patient. We report successful anesthetic management of a patient with VP undergoing distal pancreatectomy.

## Case presentation

This case report follows CARE (CAse REport) guidelines [4]. We explained the purpose of the study to the patient and obtained written informed consent to publish our findings.

A 66-year-old woman (63 kg; height, 152 cm) with VP was scheduled to undergo distal pancreatectomy for pancreatic cancer. She had been hospitalized for 3 years at the age of 28 years with abdominal pain, ileus, mental disorder, and quadriplegia. During that time, she was diagnosed with VP based on the results of several types of examination. Although she had undergone a tracheostomy due to quadriplegia during hospitalization, her neurologic symptoms disappeared after hospital discharge. Her only daughter presented with abdominal pain during her twenties, at which time she had been diagnosed with VP. She had suspected that her mother’s abdominal pain was also due to VP, although her mother had not been diagnosed with VP definitively. At the present admission, she had a history of abdominal pain that occurred every few months, which she had self-treated with sugar intake and body warming. She was also on medications for hypertension and chronic kidney disease. The creatinine level in serum was increased slightly to 2.12 mg/dL, but there were no abnormalities in other laboratory parameters, such as urinary levels of porphobilinogen and delta-aminolevulinic acid.

We selected total intravenous anesthesia (TIVA) with target-controlled infusion (TCI) as a suitable method of anesthesia [5]. In the operating theater, the upper body was covered by a forced air blanket to prevent hypothermia. General anesthesia was induced with TCI of propofol (3 μg/mL) with remifentanil (0.4 μg/kg/min) followed by rocuronium (40 mg) and tracheal intubation. An indwelling catheter was inserted into the radial artery for regular measurement of levels of glucose and electrolytes in the blood. Anesthesia was maintained with TCI of propofol and remifentanil with monitoring of the bispectral index. Rocuronium was administered intermittently to maintain a train-of-four count of 1. A total of 0.4 mg of fentanyl was administered intraoperatively, with 50 μg/h of fentanyl administered continuously for postoperative analgesia.

Blood-gas tests were undertaken via the arterial catheter every 60 min, and we checked for electrolyte abnormalities and measured blood glucose levels. All electrolytes were within the normal range intraoperatively. The blood glucose level before as well as 1, 2, 3, and 4 h after starting surgery was 102, 120, 121, 106, and 97 mg/dL, respectively. The glucose level decreased 3 h after starting surgery despite the administration of an extracellular fluid containing 5% glucose, so we administered glucose (10 g, i.v.). Body temperature decreased from 36.0 °C preoperatively to 35.5 °C 1 h after starting surgery, so we started the amino acid infusion to increase body temperature [6]. Body temperature 2, 3, and 4 h after starting the surgery was 35.4, 35.7, and 35.9 °C, respectively. The total volume of the amino acid infusion was 550 mL. Distal pancreatectomy was completed in 224 min. After confirming a train-of four count of 2, sugammadex (140 mg) was administered and the tracheal tube removed, followed by administration of oxygen (3 L/min) via a nasal cannula. She was admitted to the high dependency unit. Our patient had no neurologic abnormalities or VP symptoms. She was moved to the general ward on postoperative day 5 and was discharged from the hospital with good progress on postoperative day 15.

## Discussion

With meticulous care, we achieved safe perioperative management of a patient with VP scheduled for distal pancreatectomy. Perioperative management of porphyria is important to avoid an acute attack, and the anesthesiologist must pay careful attention to address possible triggers during this period. In this case, we focused on the selection of anesthetic drugs and prevention of hypoglycemia and hypothermia.

Porphyrias are metabolic disorders caused by altered activity of enzymes within the heme biosynthetic pathway [7]. Porphyrias can be classified as “acute” (present characteristically with neurovisceral manifestations) or “cutaneous” (present characteristically with skin lesions) [7]. Acute porphyrias include delta-aminolevulinic acid dehydratase porphyria, acute intermittent porphyria, hereditary coproporphyria, and VP. Delta-aminolevulinic acid dehydratase porphyria results in neurovisceral symptoms only. The prevalence of skin lesions increases in the order of acute intermittent porphyria, hereditary coproporphyria, and VP. VP is generated by the *PPOX* mutation, which can cause various symptoms. The most common symptom of VP is skin lesions and is observed frequently compared with other types of acute porphyria. Skin lesions are seen in 10–50% of VP patients. Our patient did not have skin lesions.

With regard to acute attacks, symptoms are, in general, non-specific and triggers vary. The common triggers are dehydration, infection, fever, fasting, hypoglycemia, and medications [8]. When our patient suffered abdominal pain, she kept herself warm and consumed sugar to prevent symptom worsening. During general anesthesia, we monitored meticulously for reddish porphyrinuria, electrolyte abnormalities, blood glucose levels, and body temperature, all of which would indicate an acute attack. If we suspected that she was developing an acute attack, we were primed to administer hemin immediately. Fortunately, there were no signs of such an attack, and hemin was not needed. Acute attacks of VP can be life-threatening unless recognized and managed appropriately. Intravenous administration of hemin is recommended if such a development should occur [9].

Selection of the most appropriate drug is highly important. Safe and dangerous drugs are listed on the Internet websites of the American Porphyria Foundation [10], the European Porphyria Network [11], and Sakuratomonokai [12] in Japan (Table 1). Some lists do not agree with others, however, and evidence is limited. In the present case, two drugs not listed for use were administered after explaining the necessity to the patient in advance: sugammadex and amino acid infusion. Sugammadex reverses neuromuscular blockade more rapidly than neostigmine and is associated with fewer adverse events [13]. Amino acid infusion has been reported to increase body temperature and improve clinical outcomes [6]. Accumulation of case reports such as this one could aid safe management of VP.

**Table 1:** Recommendations for anesthesia-related drugs in patients with porphyria. This table was created independently by the authors based on data from references [10–12]

Drug category	Safe	Probably safe	Not yet classified	Probably unsafe	Unsafe
Anesthetic	Propofol Midazolam	Desflurane	Sevoflurane		Thiopental
Analgesic	Morphine	Fentanyl Remifentanil		Pentazocine	Diclofenac Ketamine
Local anesthetic	Bupivacaine			Lidocaine	
Muscle relaxant	Rocuronium Suxamethonium	Vecuronium			
Vasopressor	Adrenaline	Phenylephrine Dopamine	Ephedrine		
Vasodilator			Diltiazem		Nifedipine
Other	Insulin	Neostigmine	Sugammadex		Phenytoin Phenobarbital

The choice of the anesthetic method merits discussion. Although the volatile anesthetic desflurane seems to be relatively safe, we selected TIVA using propofol based on previous reports [5, 14]. In addition, we did not carry out epidural anesthesia. Anesthesiologists can undertake epidural anesthesia using bupivacaine for postoperative analgesia [5, 14]. However, if neurologic symptoms occur, it might be difficult to distinguish if they are due to epidural anesthesia or porphyria. Taken together, we considered simple TIVA to be a suitable anesthetic method.

Our case report had a very important limitation: we could not obtain the details of her diagnosis. We contacted the hospital that diagnosed her and her daughter as having VP, but the data were no longer available. Nevertheless, we are confident our patient does have hereditary porphyria based on her family history.

According to CARE guidelines [4], we quote our patient directly: “I was sad when pancreatic cancer was found. Furthermore, I felt sorrow beyond that I was refused surgery at several hospitals because of porphyria. My daughter has the same disease, maybe having the same sad feeling in the future. If my surgical experience is for future patients with porphyria, I will be pleased to report my case.”

In conclusion, anesthetic management of VP patients can be undertaken safely if anesthetists pay attention to, and address, the possible triggers of acute attacks. Sugammadex and amino acid infusion may be administered safely to patients with porphyria.

## Abbreviations

*PPOX*: Protoporphyrinogen oxidase gene
TCI: Target-controlled infusion
TIVA: Total intravenous anesthesia
VP: Variegate porphyria
